# Report of a case of Cobb syndrome: multimodality imaging

**DOI:** 10.1259/bjrcr.20200145

**Published:** 2020-10-23

**Authors:** Dalia Ibrahim, Shady Mashhour

**Affiliations:** 1 Department of Radiology, Kasr Al Ainy Hospital, Cairo, Egypt

## Abstract

Cobb syndrome is a rare vascular disorder characterized by vascular skin lesions distributed in a dermatomal pattern, with corresponding muscular, osseous, paraspinal, and/or spinal vascular lesions occurring at the same body somite (metamere). We present a case of a 25-year-old man who presented with a history of right upper limb paresthesia followed by bilateral progressive upper and lower limb weakness and heaviness. Physical examination showed large cutaneous port wine stains on the right side of the chest, the nape, and along the whole right upper limb in a dermatomal distribution, with no corresponding limb hypertrophy or asymmetry. MRI and CT scan of the cervical spine showed aggressive vertebral hemangiomas involving the right side of C1 down to C4 vertebrae associated with extraosseous epidural lesion causing cervical cord compression, in addition to right paraspinal muscular low flow vascular malformations. Digital subtraction angiography of the neck vessels showed corresponding vascular blush and delayed contrast pooling in the affected regions. Cobb syndrome was diagnosed based on the dermatomal distribution of the cutaneous vascular lesions and the corresponding vertebral, epidural, and paraspinal vascular lesions occurring at the same metamere. The patient underwent a decompressive laminectomy at C2–C6 levels with removal of the epidural lesion, after which his symptoms had improved.

## Case presentation

**Figure 1.**
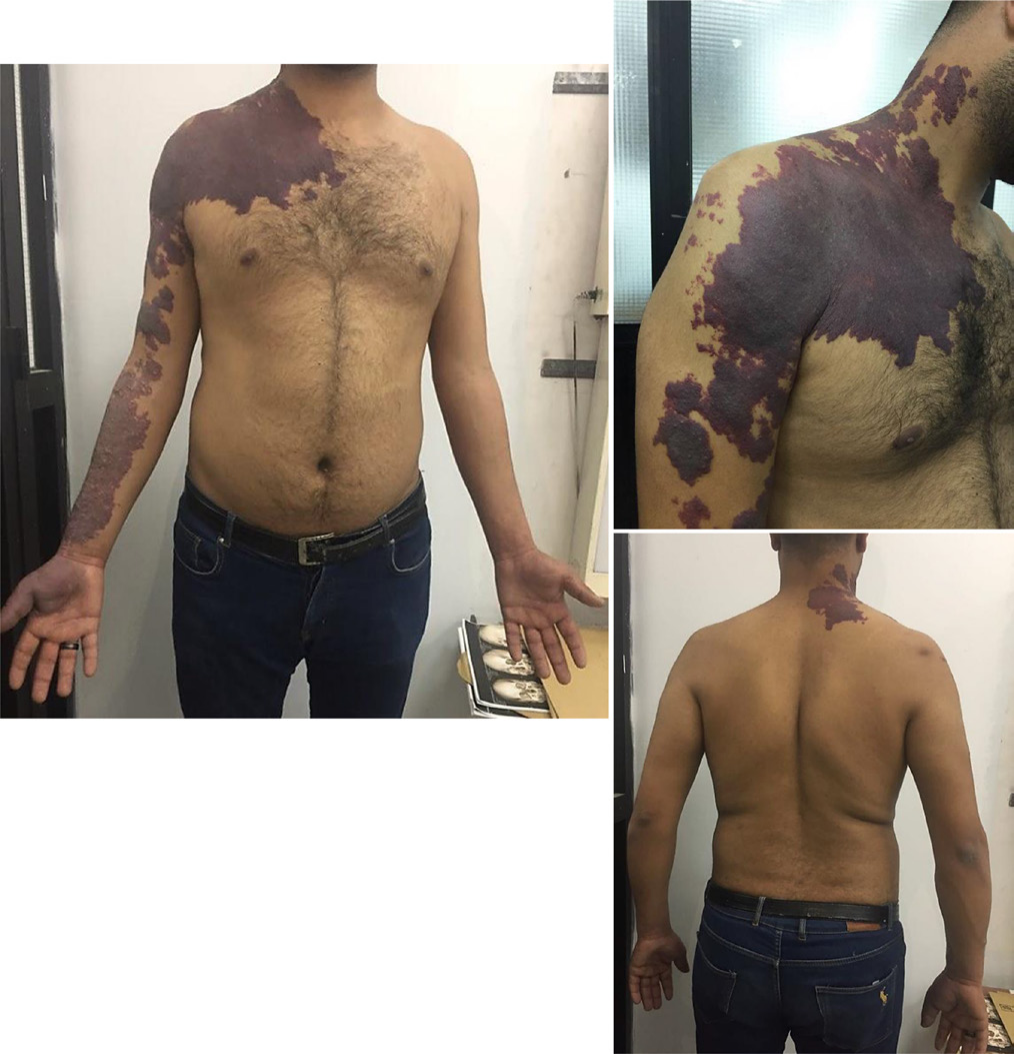
Clinical photographs of the patient demonstrating large cutaneous port wine stains involving the right side of the chest, the nape, and the whole right upper limb distal to the thumb in a dermatomal pattern.

A 25-year-old male presented with paresthesia (tingling and numbness) of the right upper limb, followed by bilateral progressive upper and lower limb heaviness and weakness evolving over a period of 3 months. Physical examination demonstrated large cutaneous port wine stains (capillary vascular malformations) involving the right side of the chest, the nape, and along the ventrolateral aspect of the whole right upper limb distal to the thumb in a dermatomal distribution, with no size discrepancy between both upper limbs (Figure 1). The cutaneous vascular malformations were present at birth and were growing proportionally with the patient without regression. The patient reported initial improvement of the port wine stains on his hand after laser treatment but the lesions later recurred. Neurologically, the patient demonstrated reduced muscle strength (Grade 3/5) of both hands and legs and decreased sensation (Grade 4/5) globally from his C3 dermatome down. He was hyperreflexic in the lower extremities. Physical examination of the rest of the body was unremarkable. No family history of similar illness.

## Investigations

**Figure 2.**
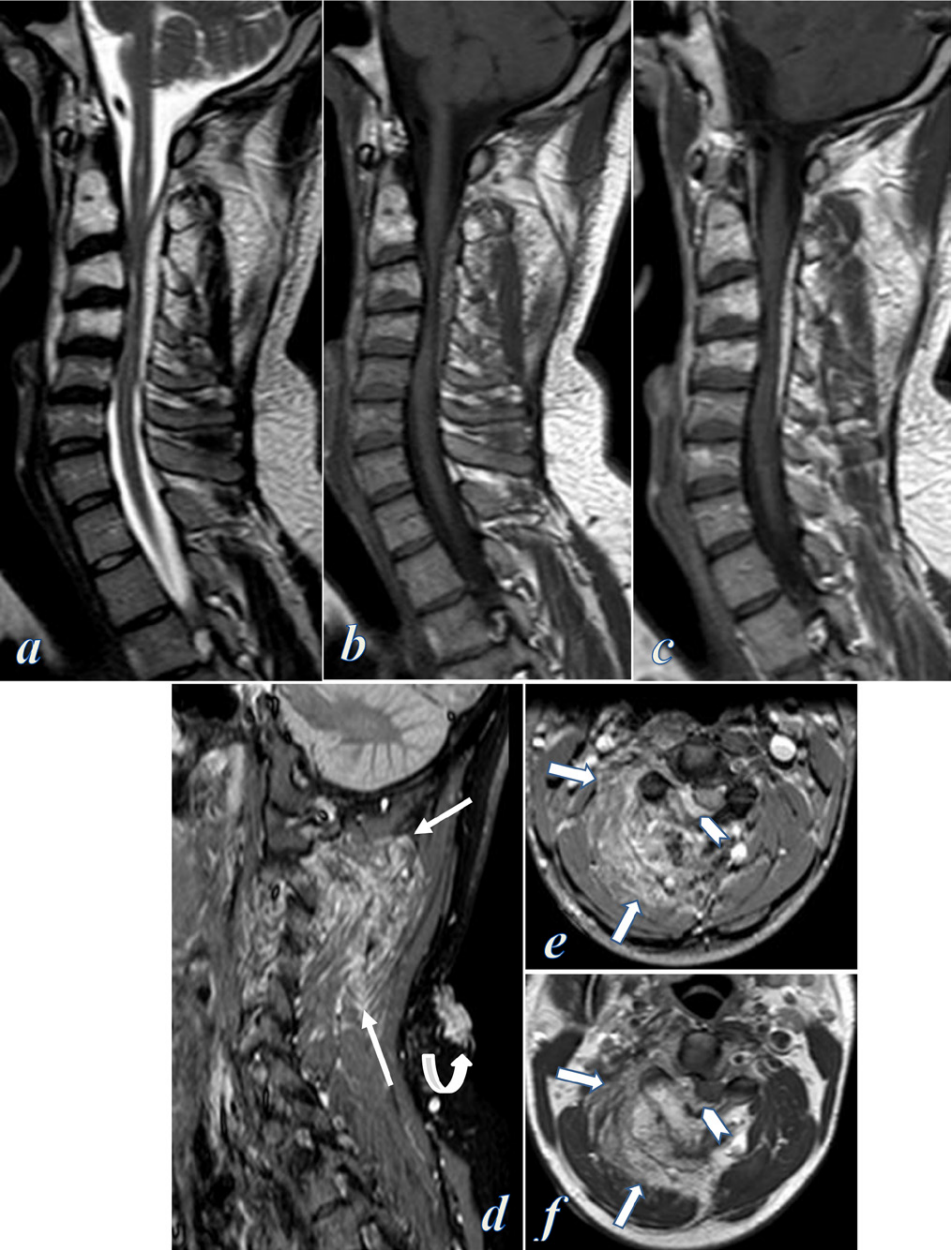
Contrast-enhanced MRI of the cervical spine; (a–c) Sagittal T2, T1 and contrast-enhanced *T*_1_ weighted MR images showing C1 down to C4 vertebral body hemangiomas eliciting high signal intensity on *T*_1_- and *T*_2_ weighted images with intense contrast enhancement, associated with extraosseous posterior epidural soft tissue lesion causing significant canal stenosis and cervical cord compression, (d) Sagittal STIR-TSE weighted image at the right parasagittal plane showed right paraspinal vascular malformations (straight arrows) and posterior cervical cutaneous vascular lesions (curved arrow), (e, f) Axial T2 and contrast-enhanced *T*_1_ weighted images at the level of C3 vertebra show bone expansion, high signal intensity and contrast enhancement of the right lamina and spinous process of C3 (vertebral hemangioma), associated with extraosseous right posterolateral epidural enhancing component (chevrons) displacing and compressing the cervical cord, and also enhancing right paraspinal vascular malformations (block arrows). STIR, short tau inversion recovery; TSE, turbo spin echo.

MRI of the cervical spine showed aggressive vertebral haemangiomas involving the right side of C1 down to C4 vertebral bodies, extending to the right pedicles, lamina, transverse and spinous processes. The vertebrae demonstrated hyperintensity on T1- and *T*_2_ weighted images, with intense contrast enhancement, associated with extraosseous posterior epidural soft tissue lesion arising between C2 and C5, more prominent on the right side, causing severe cord compression and cord edema (Figure 2a–c). MRI also revealed low flow vascular malformations involving the right paraspinal muscles infiltrating the soft tissue planes and surrounded by firbofatty stroma, eliciting intermediate signal on *T*_1_ weighted images and high signal on T2 and short tau inversion recovery (STIR) images, with no early contrast enhancement, yet they showed slow gradual diffuse enhancement on the delayed images, compatible with low flow venous vascular malformation (Figure 2).

**Figure 3.**
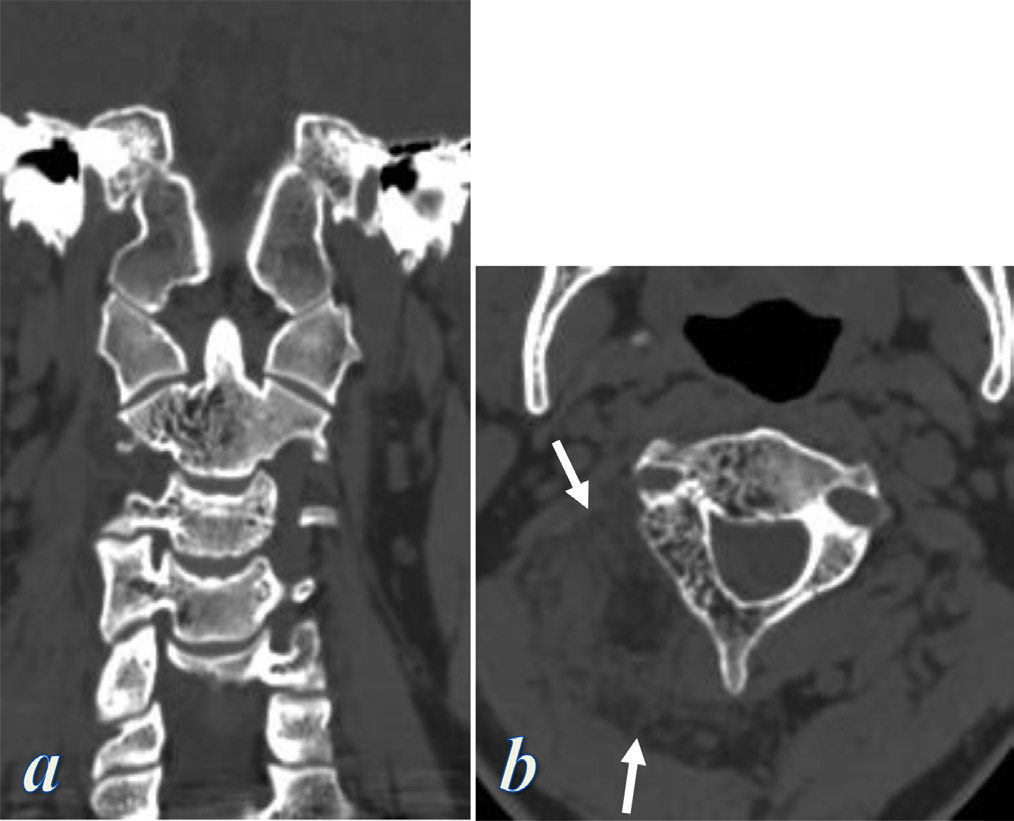
CT examination of the cervical spine; (a) Coronal reformatted CT image with bone windowing show accentuated bony trabecula involving the right side of C2 vertebra and to less extent the right transverse processes of C3 and C4 vertebrae, (b) Axial CT image with bone windowing at the level of C2 vertebra show bone expansion and accentuated trabecular markings that involve the right side of the vertebral body, right pedicle, right lamina, and spinous process, giving the typical “polka dot” sign. Note also the right paraspinal vascular lesions (arrows in b).

CT scan of the involved vertebrae demonstrated typical features of osseous hemangiomas including bone expansion and accentuation of the trabecular bone markings, forming the pathognomonic “polka–dot” sign (Figure 3).

**Figure 4.**
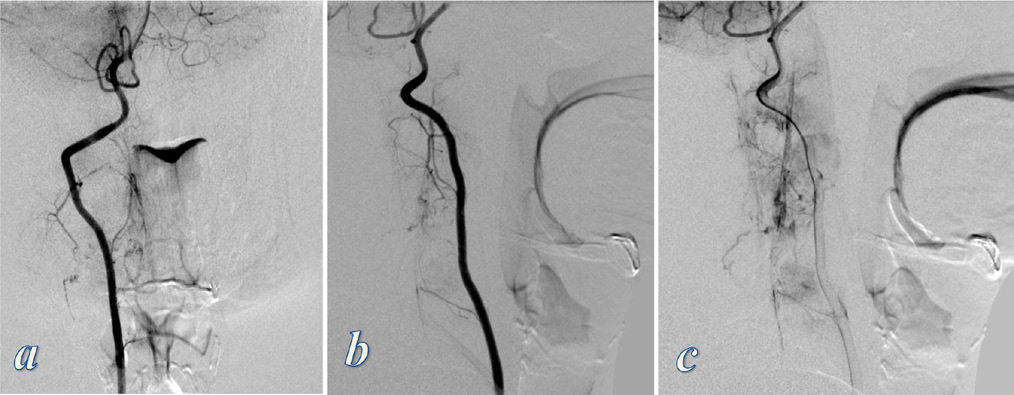
DSA of the right vertebral artery; (a) anteroposterior view arterial phase, (b, c) lateral views; arterial and delayed phases. Images show vascular blush and delayed contrast pooling within the affected regions supplied by the hypertrophied spinal and muscular branches of the right vertebral artery. No evidence of high flow arteriovenous communications. DSA, digital subtraction angiography.

Digital subtraction angiography (DSA) of the vertebral vessels showed corresponding vascular blush and delayed contrast pooling within the affected vertebrae and surrounding soft tissue, supplied mainly by the hypertrophied spinal and muscular branches of the foraminal segment (V2) of the right vertebral artery (Figure 4). No evidence of high flow arteriovenous communications.

**Figure 5.**
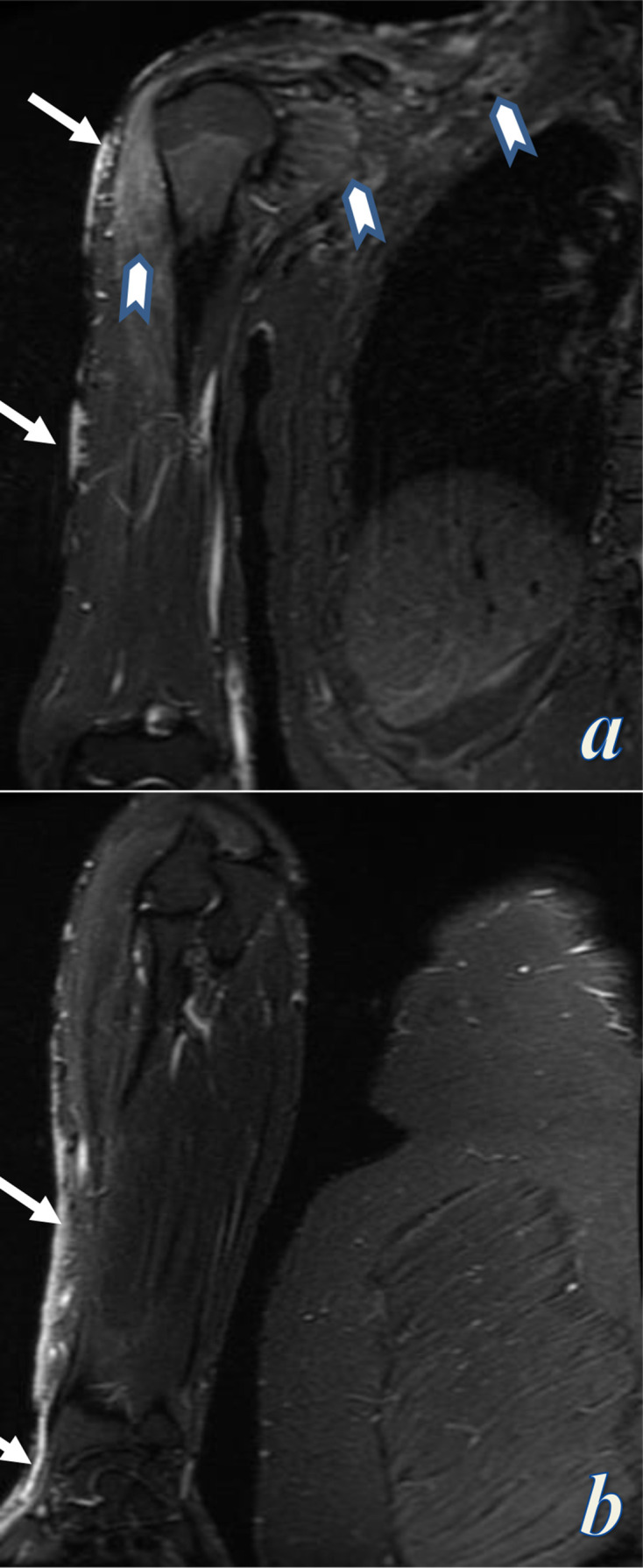
MRI of the right upper limb; Coronal STIR-weighted images of the right arm (a) and the right forearm (b). Images demonstrate cutaneous vascular malformations along the lateral aspect of the shoulder, arm, forearm, distal to the thumb (white arrows), associated with deep low flow vascular malformations involving the right paraspinal, subscapularis, and deltoid muscles eliciting bright signal on STIR images and surrounded by edema signal and fibrofatty stroma (chevrons in a). STIR, short tau inversion recovery.

MRI of the right upper limb demonstrated cutaneous vascular malformations on the shoulder and the ventrolateral aspect of the right upper limb eliciting bright signal on STIR images. MRI also showed low flow vascular malformations involving the right paraspinal, subscapularis, teres minor, and deltoid muscles infiltrating through the adjacent fat planes and surrounded by edema and fibrofatty stroma eliciting intermediate signal on *T*_1_ weighted images and high signal on *T*_2_ weighted and STIR images, and showing diffuse gradual delayed contrast enhancement (Figure 5). The superficial and deep venous systems were intact with no evidence of venous ectasia or varicosities. No bony or soft tissue hypertrophy.

## Treatment

**Figure 6.**
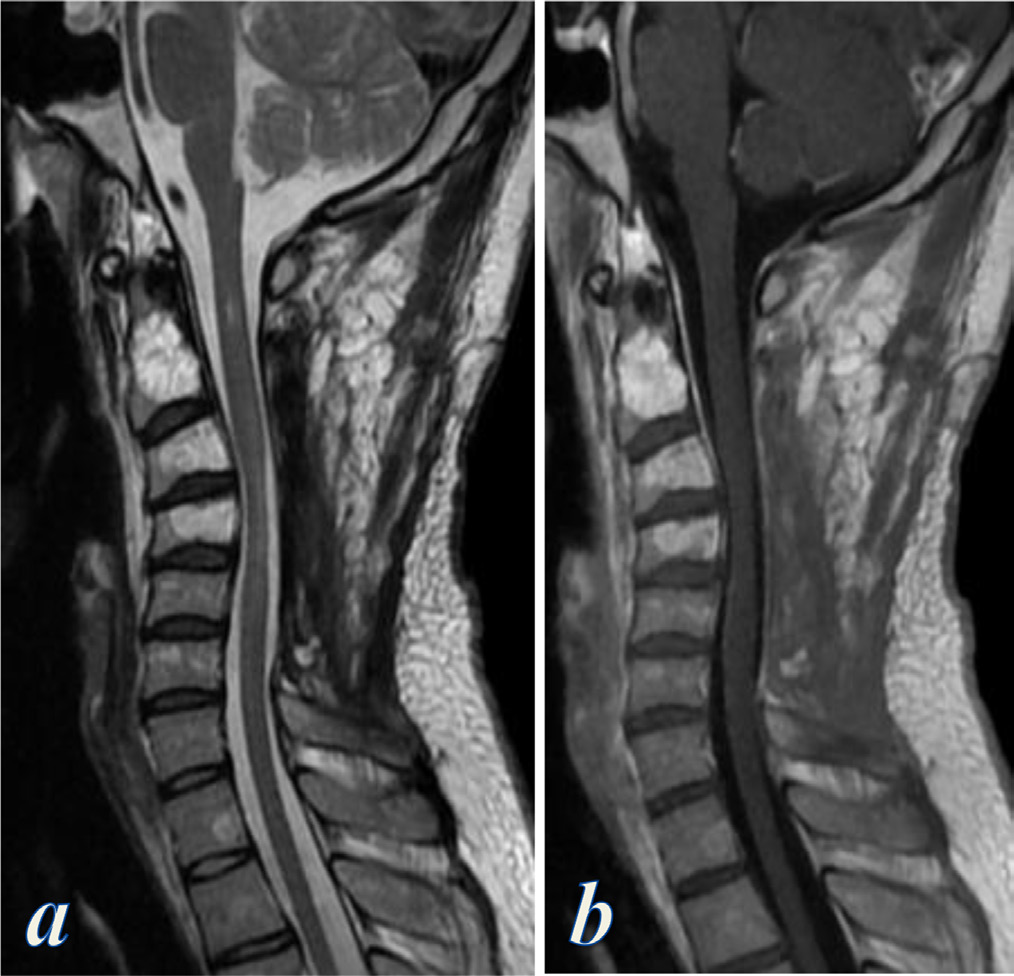
Post-operative contrast-enhanced MRI of the cervical spine; (a, b) Sagittal T2 and contrast-enhanced *T*_1_ weighted MR images show C2–C6 laminectomy with surgical excision of the epidural component and adequate decompression of the spinal canal. The cervical cord opposite C4 vertebra appears thinned and shows mild increased intramedullary signal, representing compressive cord myelopathy.

Given the rapidity of the neurological decline and severe spinal cord compression, surgical intervention was elected for treatment. The patient underwent decompressive bilateral laminectomy at C2–C6 levels with excision of the epidural lesion, which appeared highly bloody at surgery (Figure 6). Histological examination of the epidural lesion revealed a lesion formed of large vascular spaces showing flat endothelial lining with intraluminal red blood cells, without atypia or mitosis, associated with pieces of bony tissue, consistent with cavernous hemangioma partially involving bone. The patient had an uneventful recovery and demonstrated improvement in motor strength, progressing to Grade 4/5 in his extremities bilaterally.

## Discussion

Cobb syndrome, also known as spinal arteriovenous metameric syndrome or cutaneomeningospinal angiomatosis, is a rare neurocutaneous vascular disorder characterized by combined cutaneous, muscular and/or bony vascular lesions as well as spinal or paraspinal vascular lesions involving the same body somite (metamere).^1^

The original description of the syndrome was the association of the spinal and cutaneous angiomas by Cobb in 1915.^2^ However, conceptually, it is not necessarily required to have both cutaneous and spinal vascular malformations. Any combination of vascular malformations in the same metameres are possible, even if the lesions are not involving the spinal cord.^3,4^

The disease is genetic, non-hereditary, and is believed to be due to a sporadic mutation of the mother cells at an early stage of embryogenesis resulting in multiple vascular malformations in parts or all tissues of the same somatomeric distribution, including the spine, muscles, skeleton, soft tissues, and skin.^4^

Though the disease is present since birth, clinical manifestations are often not seen until later in life. Patients typically present with sudden onset of pain, weakness, paralysis, or paresthesia in the extremities that can be localized below a specific dermatome.^4^ The pathogenesis of neurological symptoms in Cobb syndrome is believed to be due to a variety of factors, including cord compression by the vascular malformations, blood steal syndrome resulting in cord ischemia, and venous hypertension.^3,4^

The cutaneous vascular lesions are present at birth and don’t tend to resolve spontaneously. They range from macular port wine stains to raised papular vascular lesions like angiomas, angiokeratomas, angiolipomas, and lymphangioma circumscriptum. The dermatomal distribution of the cutaneous lesions is important, as this may raise the suspicion of Cobb syndrome.^5^

The deep vascular lesions occurring at the same body metamere can be within the spinal cord itself (intramedullary), outside the cord (intraspinal extramedullary), vertebral or extraspinal, including the paraspinal soft tissues.^5^ Under the new International Society for the Study of Vascular Anomalies classification system, vascular lesions which were previously described as Cobb syndrome would now be better characterized as vascular malformations rather than true hemangiomas. Cobb syndrome is usually associated with spinal high-flow vascular malformations; spinal arteriovenous vascular malformations are classified into four subtypes, Cobb Syndrome is associated with Type 3 arteriovenous malformations.^6^ In our case, however, there was no spinal cord involvement, and the extra medullary vascular lesions were all of the low-flow type. Though the majority of spinal vascular lesions in Cobb syndrome represent high flow vascular malformations, spinal low-flow vascular malformations associated with cutaneous vascular lesions occurring in the same metamere are also defined as Cobb syndrome.^7–9^

Diagnosis of Cobb syndrome occurs when patients present with three or more of the following five factors: (1) spinal intramedullary vascular malformation, (2) intraspinal epidural venous vascular malformations, (3) vertebral osseous hemangioma, (4) paravertebral vascular malformations, or (5) cutaneous/subcutaneous vascular malformations.^10^ Our patient fulfilled four of the five criteria including vertebral, epidural, paraspinal muscular, and cutaneous vascular malformations.

Radiological examinations are important tools for the diagnosis of Cobb syndrome. CT/CT angiography and MRI/MR angiography are useful modalities to identify deep vascular malformations.^11^ MRI/MR angiography aids to detect precisely full extensions of the spinal vascular lesions. In case of arteriovenous fistula, there are multiple dilated and tortuous flow void perimedullary vessels seen on *T*_2_ weighted images, the serpentine vascular structures are best appreciated on heavily *T*_2_ weighted sequences [constructive interference in steady-state), fast imaging employing steady-state acquisition ), or 3D turbo spin-echo)] compared with standard T2 TSE sequences.^12^ Contrast-enhanced MRA is useful in localizing spinal dural fistula. The AV shunt is usually confirmed by the first-pass gadolinium-enhanced technique which demonstrates the early venous filling and the level of the shunt.^13^ MRI also demonstrates spinal cord signal changes, like intramedullary edema and diffuse cord enhancement secondary to cord congestion/ischemia.^14^

DSA is essential for a definite diagnosis. It’s the gold-standard modality in localizing and defining the full extent of spinal vascular lesions. It also helps to plan for treatment strategy and to allow occlusion via embolization.^15^

Treatment decision is guided by the patient’s symptoms and imaging features; treatment of osteomuscular malformations involves endovascular embolization with occlusion of the feeding arteries and/or surgery. Treatment of spinal vascular lesions involves the use of steroids, surgery, and endovascular embolization. Surgery is usually indicated in cases of rapid/progressive neurological symptoms, pre-operative embolization is used to reduce intraoperative bleeding of these hypervascular lesions.^16^

Early diagnosis of Cobb syndrome is important as it allows rapid treatment, minimizing future neurological deficits especially paralysis or sensory deficits. Recognition of cutaneous vascular lesions with dermatomal distribution should prompt further evaluation for underlying spinal vascular lesions.^17^

## Learning points

1. Cobb syndrome is a rare non-hereditary, genetic, vascular disorder.
2. Cobb syndrome is characterized by cutaneous vascular lesions distributed in a dermatomal pattern, associated with underlying muscular, bony, intraspinal, or paraspinal vascular malformations involving the same body somite (metamere).
3. Recognition of cutaneous vascular lesions with dermatomal distribution may hint underlying spinal vascular malformations which should prompt further evaluation and treatment in order to prevent future neurological deficits.
4. MR imaging and digital subtraction angiography are important diagnostic tools.
5. Treatment options include endovascular embolization and surgical excision.

## References

[1] Elkordy A, Endo T, Sato K, Sonoda Y, Takahashi A, Tominaga T Exclusively epidural spinal metameric arteriovenous shunts: case report and literature review. Spine J 2015; 15: e15–22. doi: 10.1016/j.spinee.2014.11.022 25450654

[2] Cobb S Haemangioma of the spinal cord: associated with skin naevi of the same metamere. Ann Surg 1915; 62: 641–9. doi: 10.1097/00000658-191512000-00001 17863459 PMC1406820

[3] Komiyama M, Ishiguro T, Terada A, Watanabe Y, Nakajima H, Ohata Y, et al Spinal arteriovenous metameric syndrome in a neonate presenting with congestive heart failure: case report. Childs Nerv Syst 2014; 30: 1607‐–11. doi: 10.1007/s00381-014-2439-y 24845229

[4] Choi IS Spinal arteriovenous metameric syndrome: angioarchitecture and their prognosis. AJNR Am J Neuroradiol 2013; 34: 464–5. doi: 10.3174/ajnr.A3318 22936097 PMC7965121

[5] Pal P, Ray S, Chakraborty S, Dey S, Talukdar A Cobb syndrome: a rare cause of paraplegia. Ann Neurosci 2015; 22: 191–3. doi: 10.5214/ans.0972.7531.220312 26130930 PMC4481555

[6] Nozaki T, Nosaka S, Miyazaki O, Makidono A, Yamamoto A, Niwa T, et al Syndromes associated with vascular tumors and malformations: a pictorial review. Radiographics 2013; 33: 175–95. Jan-Feb. doi: 10.1148/rg.331125052 23322836

[7] Clinton TS, Cooke LM, Graham BS Cobb syndrome associated with a verrucous (angiokeratomalike) vascular malformation. Cutis 2003; 71: 283–7. 12729091

[8] Johnson WD, Petrie MM Variety of spinal vascular pathology seen in adult cobb syndrome. J Neurosurg Spine 2009; 10: 430–5. doi: 10.3171/2009.1.SPINE08334 19442004

[9] Lee EJ, Kang SW, Shinn KS Cobb’s syndrome: a case report. J Korean Radiol Soc 1997; 36: 33–6. doi: 10.3348/jkrs.1997.36.1.33

[10] Wan L, Ge W-R, Shi X-Y, Wang J, Hu L-Y, Zou L-P, et al Cobb syndrome manifesting as repetitive seizures in a 10-year-old girl: a case report and literature review. Front Neurol 2019; 10: 1302. doi: 10.3389/fneur.2019.01302 31866938 PMC6910015

[11] Saraf-Lavi E, Bowen BC, Quencer RM, Sklar EML, Holz A, Falcone S, et al Detection of spinal dural arteriovenous fistulae with MR imaging and contrast-enhanced Mr angiography: sensitivity, specificity, and prediction of vertebral level. AJNR Am J Neuroradiol 2002; 23: 858‐–67. 12006294 PMC7974721

[12] Krings T, Geibprasert S Spinal dural arteriovenous fistulas. AJNR Am J Neuroradiol 2009; 30: 639–48. doi: 10.3174/ajnr.A1485 19213818 PMC7051782

[13] Farb RI, Kim JK, Willinsky RA, Montanera WJ, terBrugge K, Derbyshire JA, et al Spinal dural arteriovenous fistula localization with a technique of first-pass gadolinium-enhanced MR **angiography: initial experience**. Radiology 2002; 222: 843–50. doi: 10.1148/radiol.2223010826 11867811

[14] Gilbertson JR, Miller GM, Goldman MS, Marsh WR Spinal dural arteriovenous fistulas: Mr and myelographic findings. AJNR Am J Neuroradiol 1995; 16: 2049–57. 8585493 PMC8337217

[15] Miyatake S-ichi, Kikuchi H, Koide T, Yamagata S, Nagata I, Minami S-suke, et al Cobb’s syndrome and its treatment with embolization. J Neurosurg 1990; 72: 497–9. doi: 10.3171/jns.1990.72.3.0497 2406383

[16] Linfante I, Tari Capone F, Dabus G, Gonzalez-Arias S, Lau PE, Samaniego EA Spinal arteriovenous malformation associated with spinal metameric syndrome: a treatable cause of long-term paraplegia? J Neurosurg Spine 2012; 16: 408‐–13. doi: 10.3171/2011.12.SPINE11636 22225485

[17] Soeda A, Sakai N, Iihara K, Nagata I Cobb syndrome in an infant: treatment with endovascular embolization and corticosteroid therapy: case report. Neurosurgery 2003; 52: 711–5. doi: 10.1227/01.NEU.0000048483.21777.B7 12590699

